# Sociospatial distribution of access to facilities for moderate and vigorous intensity physical activity in Scotland by different modes of transport

**DOI:** 10.1186/1479-5868-9-55

**Published:** 2012-07-03

**Authors:** Karen E Lamb, David Ogilvie, Neil S Ferguson, Jonathan Murray, Yang Wang, Anne Ellaway

**Affiliations:** 1 Medical Research Council Social and Public Health Sciences Unit, 4 Lilybank Gardens, Glasgow, G12 8RZ, Scotland, UK; 2 Medical Research Council Epidemiology Unit and UKCRC Centre for Diet and Activity Research (CEDAR), Box 296, Institute of Public Health, Forvie Site, Robinson Way, Cambridge, CB2 0SR, UK; 3 Department of Civil Engineering, University of Strathclyde, John Anderson Building, 107 Rottenrow, Glasgow, G4 0NG, Scotland, UK

**Keywords:** Deprivation, Accessibility, Intensity, Recreational physical activity, Transport

## Abstract

**Background:**

People living in neighbourhoods of lower socioeconomic status have been shown to have higher rates of obesity and a lower likelihood of meeting physical activity recommendations than their more affluent counterparts. This study examines the sociospatial distribution of access to facilities for moderate or vigorous intensity physical activity in Scotland and whether such access differs by the mode of transport available and by Urban Rural Classification.

**Methods:**

A database of all fixed physical activity facilities was obtained from the national agency for sport in Scotland. Facilities were categorised into light, moderate and vigorous intensity activity groupings before being mapped. Transport networks were created to assess the number of each type of facility accessible from the population weighted centroid of each small area in Scotland on foot, by bicycle, by car and by bus. Multilevel modelling was used to investigate the distribution of the number of accessible facilities by small area deprivation within urban, small town and rural areas separately, adjusting for population size and local authority.

**Results:**

Prior to adjustment for Urban Rural Classification and local authority, the median number of accessible facilities for moderate or vigorous intensity activity increased with increasing deprivation from the most affluent or second most affluent quintile to the most deprived for all modes of transport. However, after adjustment, the modelling results suggest that those in more affluent areas have significantly higher access to moderate and vigorous intensity facilities by car than those living in more deprived areas.

**Conclusions:**

The sociospatial distributions of access to facilities for both moderate intensity and vigorous intensity physical activity were similar. However, the results suggest that those living in the most affluent neighbourhoods have poorer access to facilities of either type that can be reached on foot, by bicycle or by bus than those living in less affluent areas. This poorer access from the most affluent areas appears to be reversed for those with access to a car.

## Background

Obesity is a major public health concern worldwide, with 1.46 billion people estimated to be overweight and approximately 500 million estimated to be obese [1]. In Scotland, approximately 70% of men and 60% of women are overweight, with over 25% of adults estimated to be obese [2]. Studies have shown a higher risk of obesity and lower levels of physical activity among more disadvantaged groups [3-6]. Despite recommendations that adults should participate in at least 30 minutes of moderate intensity physical activity (PA) each day, fewer than half achieve this [2] with levels being particularly low among women from deprived areas [7].

Since efforts to increase PA levels focussed on changing individuals’ behaviour have had limited success, attention has turned towards examining the extent to which local environmental factors, such as access to PA facilities, differ between more affluent and deprived areas. A national study conducted in the USA found that areas with higher socioeconomic status had greater access to physical fitness facilities and membership based sports clubs [8], whilst in New Zealand sports facilities were found to be more accessible from the more deprived areas [9]. In a study of English sports facilities, Hillsdon et al. found fewer facilities in poorer areas [10]. Other studies, mainly conducted outside the UK, have shown conflicting patterns of accessibility [5,11-14]. In our previous analysis, although we found a statistically significant association between area-level income deprivation and the number of all, public and private physical activity facilities in Scotland, there was no clear pattern in the relationship [15]. In addition, we assessed the differences in the number of accessible facilities within specified walking and cycling thresholds by deprivation [16] and found that access to facilities was generally lower for those living in more affluent areas than for those living in the other quintiles of deprivation. In subsequent analysis, we found that this was also the case for access by bus in urban areas; for access by car, on the other hand, in rural areas those living in more affluent areas had significantly higher access than those living in more deprived areas [17].

It may be that in examining the presence or otherwise of facilities *per se*, important differences in the nature of local facilities may be masked. One important dimension to consider is that different people may be attracted to, or able to participate in, activities of different physical intensities. In this paper, we therefore categorise facilities according to the opportunity they offer for participation in activities of light, moderate or vigorous intensity and examine whether the accessibility of these different types of facility differs by area deprivation and by Urban Rural Classification. We adjust for Urban Rural Classification to take into account the proximity to population centres which potentially contain a variety of different facilities. To our knowledge, no other studies have examined the differential accessibility of facilities classified in this way. We also directly compare, for the first time, how the sociospatial patterning of accessibility to facilities differs between four alternative modes of transport that may be used to reach them.

## Methods

### Physical activity facilities

The national agency for sport in Scotland, **sport**scotland, supplied a list of the names, types and the British National Grid Reference [18] of all fixed indoor and outdoor PA facilities in Scotland [19]. Duplicate facilities of the same type were omitted from the dataset prior to the analysis, as documented in our previous paper [15]. Each of the PA facilities, which consisted of amenities such as swimming pools, football pitches and golf courses, was assigned a typical energy expenditure value according to the metabolic equivalent of task (MET) intensity value from the compendium of Ainsworth et al*.*[20]. One MET is defined as the energy cost of a person at rest. The compendium provides a comprehensive list of activities and their corresponding MET values and was created to enable a consistent classification of energy expenditure from self-report activity data across studies. Standard cut-points defined by Pate et al. [21] of less than 3 METs for light intensity activity, 3 to 6 METs for moderate intensity activity and greater than 6 METs for vigorous intensity activity were adopted and the facilities were grouped into light, moderate and vigorous intensity categories according to the typical activity which was assumed to be undertaken at the facility. Light intensity activities include, for example, hunting with a bow and arrow and pistol shooting which both have MET values of 2.5. Facilities such as ballistics halls were therefore grouped in the light category. Moderate intensity activities consist of those which perhaps in principle could involve an average MET of greater than 6 but for which this value would be atypical and include golf (compendium values for all golfing activities are less than 5 METs), bowling (3 METs) and cricket (5 METs). Facilities such as golf courses, bowling greens and cricket squares were therefore grouped in the moderate intensity category. Athletics tracks are commonly used for hard training purposes and a MET of greater than 6 is within the limits of normal usage of a swimming pool. Facilities such as athletics tracks and swimming pools were therefore classified as vigorous intensity facilities. Activities such as shinty (a team sport mainly played in the Highlands of Scotland) and Gaelic football, which did not feature in the compendium, were classed as vigorous as these activities would typically be performed at vigorous intensity. Some fixed PA facilities, such as occasional sports halls and church halls, were difficult to match to a particular activity from the METs compendium. It was assumed that in practice people were most likely to participate in activities such as badminton and table tennis (moderate intensity activities) in these spaces. These facilities were therefore assigned to the moderate intensity classification. Of the 10,032 fixed PA facilities, only 10 (0.1%) — comprising ballistics halls, croquet lawns and indoor small bore rifle ranges — were classified as light intensity facilities. This category of facilities was therefore not considered in subsequent analysis. 3,872 (38.6%) were classified as facilities in which, in general, moderate intensity activity would be conducted and 6,150 (61.3%) were classified as facilities in which vigorous intensity activity would predominantly be undertaken.

### Transport network

The PA facilities were mapped using Geographic Information System (GIS) software and the number of light, moderate and vigorous intensity activity facilities accessible, with accessible defined to be within a 20 or 30 minute journey, on foot, by bicycle, by car and by bus from the population weighted centroid of each datazone (DZ) was calculated, as detailed below.

DZs are the key small area measure in Scotland and are created from groups of output areas in the 2001 Census [22] and are nested within Scottish local authorities. DZs were created to respect natural boundaries and communities and consist of households with similar socioeconomic characteristics. There are 6,505 DZs in Scotland with a mean population of 778 individuals (range 476–2813) and mean area of 11.9km^2^. The most important criterion adopted in the initial definition of DZs was to have roughly equal population sizes of between 500 and 1,000 individuals and so the DZs are variable in terms of the area they cover. The smallest DZ is located in Edinburgh and covers an area of only 12,367m^2^ whilst the largest data zone is located in the Highlands and covers an area of 1,159km^2^. 6,412 (98.6%) of the 6,505 DZs are located within mainland Scotland local authorities. The Western Isles, Shetland and Orkney Islands were not considered in this analysis as the transport network did not include these island local authorities.

A transport network was created using TransCAD software version 5.0 [23] which combines a GIS with transport planning functionality. Data from the Ordnance Survey Integrated Transport Network layer covering mainland Scotland were imported into TransCAD. In addition, the population weighted centroids of DZs and the locations of the PA facilities were imported into TransCAD, with dummy links added to connect these point features to the nearest node on the Integrated Transport Network layer.

For walking and cycling, routes in which these modes are not possible (such as motorways) were removed. An average walking speed of 5km/hr [24] and an average cycling speed of 14km/hr [25] were assumed. Considering the car and bus transport networks, the car network was created to represent uncongested road conditions, with the estimated free flow speed of the road type (motorway, A road, B road, minor road, local street) adopted as the travel speed in the analysis. Time penalties were allocated to left and right turning traffic movements at junctions to reflect the delay experienced by vehicles negotiating the geometry of the junction in accordance with values estimated by McDonald et al. [26]. Further details of the car and bus network adopted in this analysis are documented elsewhere [17].

Bus routes were created using bus timetable information obtained from the National Public Transport Data Repository [27]. This dataset contained details of all bus stop locations and scheduled bus journeys in Scotland during a selected week in October 2007. The bus schedule data were examined and routes which followed a common sequence of stops were identified, resulting in 12,371 unique routes. Each route was checked for errors in either the original data or the route creation process to verify the route system. In this analysis, bus routes operating between 10 am and 4 pm on a Wednesday were selected to represent bus services on a weekday inter-peak period. For this period a bus network was created which incorporated the surrounding road network for access, egress and interchange trip stages.

A matrix of travel times between the population weighted centroid and each of the PA facilities was determined for each mode of transport assuming that the travellers would take the shortest possible path by distance for walking and cycling and by time for bus and car travel. Population weighted centroids were chosen as this is the point in the DZ which minimises the distance for all of the households in the area [28]. For bus networks, the maximum number of transfers between bus services was restricted to two and the maximum access and egress walk times were limited to 30 minutes. The bus stop waiting times were allowed to be no greater than five minutes, under an assumption that passengers would know the bus timetable and ensure an arrival time at the bus stop which would avoid excessive waiting times. Each matrix was then used to determine the number of moderate and vigorous intensity activity facilities accessible within 20 and 30 minute thresholds from the population weighted centroid of each DZ for each mode of transport.

### Data zone level variables

Three publicly available DZ measures were considered in the analysis of the distribution of facilities in Scotland. The 2006 Scottish Index of Multiple Deprivation (SIMD) Current Income sub-domain [29] was obtained in order to explore the distribution of accessibility to facilities by small area deprivation. The SIMD provides a measure of compound social and material deprivation and is calculated using data on education, employment, welfare benefits, health, housing and other population characteristics for each DZ. The full SIMD contains information about access to services which would perhaps introduce a degree of circularity into an analysis looking at the accessibility of PA facilities. We therefore used the Current Income sub-domain and grouped the continuous measure into quintiles ranging from the most affluent DZs in quintile 1 to the most deprived DZs in quintile 5. The Scottish Executive six-fold Urban Rural Classification [30] and the 2001 Census population numbers [31] were also acquired for adjustment in the analysis. The Urban Rural Classification consists of three types of area; urban areas (category 1=large urban areas, category 2=other urban areas), small towns (category 3=accessible small towns, category 4=remote small towns) and rural areas (category 5=accessible rural areas, category 6=remote rural areas), with mean areas of 0.5km^2^, 2.4km^2^ and 62.1km^2^ respectively. The six-fold Urban Rural Classification defines areas according to population size as well as drive time to the nearest urban area. Large urban areas consist of areas with over 125,000 residents whilst other urban areas are those with between 10,000 and 125,000 residents. Small towns, both accessible and remote, consist of areas with between 3,000 and 10,000 residents with accessible small towns defined to be within a 30 minute drive of a settlement of an urban area and remote small towns defined to have more than a 30 minute drive to an urban area. Rural areas have fewer than 3,000 residents with accessible rural areas defined to be within a 30 minute drive of an urban area and remote rural areas defined to have more than a 30 minute drive to an urban area.

### Statistical analysis

The median, minimum and maximum number of moderate and vigorous intensity activity facilities accessible by each mode of transport within 20 and 30 minute thresholds from the population weighted centroid was calculated for each Current Income sub-domain SIMD quintile.

Multilevel negative binomial regression was used to model the relationship between the number of accessible facilities and Income SIMD for each sub-category of facility type and each time threshold separately, taking into account the hierarchical structure with DZs located within local authorities. Significant interaction effects were identified between Urban Rural Classification and Income SIMD. Therefore, separate models were fitted for the urban, small town and rural areas to aid interpretation of the results. A poisson multilevel model was adopted when modelling the moderate intensity activity facilities accessible within a 20 minute walk of small towns as no overdispersion was found to be present for this particular outcome.

Although multilevel modelling allows for the clustering of DZs within local authorities, it does not take into account the spatial location of the DZs. Spatial data are often affected by positive spatial correlation by which areas near one another have more attributes in common with each other than with areas located further away [32]. If spatial autocorrelation is not taken into account in the modelling, the resulting parameter estimates may be biased.

Using the approach adopted in our previous paper [16], the Moran’s I permutation test was carried out to test for the presence of spatial autocorrelation. This tests the null hypothesis of no spatial autocorrelation between DZs sharing a common border [33,34]. Where statistically significant positive spatial autocorrelation was present, a spatial weighting variable dependent on the response variable in each model was included in the regression to take account of the spatial location of the DZs, as documented in a previous analysis [16]. The spatial variable was not adopted in the modelling of the number of moderate intensity facilities accessible within a 20 minute bus journey of urban areas or the number of vigorous intensity facilities accessible within a 20 minute cycle of rural areas as it was not found to reduce the residual spatial autocorrelation.

Even after the inclusion of the spatial weighting variable, where appropriate, statistically significant residual spatial autocorrelation remained. For this reason, a more conservative 99% level of significance was used.

The statistical analysis was carried out using R version 2.11.1 [35]. The modelling results are presented as graphs of the rate ratio (RR) of accessible facilities with 99% confidence intervals. The most affluent Income SIMD quintile was the baseline category in the modelling. A rate ratio of greater than one indicates higher accessibility than the most affluent quintile.

## Results

### Distribution of facilities by area-level deprivation

Table 1 shows the median, minimum and maximum number of moderate and vigorous intensity activity facilities accessible within a 20 minute journey by Income SIMD for each transport mode. Considering facilities for moderate intensity activities, the median number of accessible facilities by bicycle and car, both unadjusted and population adjusted, increased as level of deprivation increased from the second most affluent quintile (Q2) to the most deprived quintile (Q5). By bus, the median number, after adjustment for population, increased from Q1 to Q5 whilst on foot the increase was from Q1 to Q4 for the population adjusted figures.

**Table 1:** Median, minimum and maximum number of accessible facilities within 20 minutes by mode of transport and deprivation

**MODERATE INTENSITY FACILITIES**	**WALKING**		**CYCLING**		**BUS**		**CAR**	
**Income deprivation quintile**	Unadjusted	Adjusted	Unadjusted	Adjusted	Unadjusted	Adjusted	Unadjusted	Adjusted
1 (most affluent)	2.0 (0.0, 33.0)	3.2 (0.0, 48.2)	18.0 (0.0, 199.0)	23.2 (0.0, 318.7)	7.0 (0.0, 248.0)	9.0 (0.0, 354.6)	376.0 (0.0, 839.0)	481.2 (0.0, 1440.2)
2	2.0 (0.0, 34.0)	3.2 (0.0, 52.6)	12.0 (0.0, 206.0)	14.7 (0.0, 337.3)	7.0 (0.0, 281.0)	9.3 (0.0, 435.6)	196.0 (0.0, 842.0)	259.8 (0.0, 1458.0)
3 (middling)	3.0 (0.0, 36.0)	4.2 (0.0, 47.9)	14.0 (0.0, 205.0)	1 7.8 (0.0, 323.1)	9.0 (0.0, 256.0)	11.4 (0.0, 361.1)	226.0 (0.0, 842.0)	306.8 (0.0, 1591.2)
4	4.0 (0.0, 32.0)	5.2 (0.0, 54.1)	21.0 (0.0, 201.0)	26.9 (0.0, 352.8)	13.0 (0.0, 242.0)	16.7 (0.0, 442.4)	367.0 (2.0, 838.0)	480.2 (2.7, 1439.4)
5 (most deprived)	4.0 (0.0, 30.0)	4.8 (0.0, 56.5)	28.0 (1.0, 166.0)	35.9 (1.0, 291.9)	15.0 (0.0, 179.0)	19.2 (0.0, 295.7)	564.0 (7.0, 823.0)	648.4 (8.1, 1461.4)
**VIGOROUS INTENSITY FACILITIES**								
**Income deprivation quintile**								
1 (most affluent)	4.0 (0.0, 45.0)	5.7 (0.0, 58.4)	33.0 (0.0, 242.0)	43.8 (0.0, 396.4)	12.0 (0.0, 284.0)	15.3 (0.0, 393.9)	633.5 (0.0, 1643.0)	818.8 (0.0, 3061.9)
2	4.0 (0.0, 47.0)	5.1 (0.0, 69.2)	19.0 (0.0, 257.0)	25.3 (0.0, 408.7)	10.0 (0.0, 348.0)	13.3 (0.0, 530.3)	336.5 (0.0, 1639.0)	450.8 (0.0, 2958.3)
3 (middling)	6.0 (0.0, 34.0)	7.7 (0.0, 54.6)	26.0 (0.0, 250.0)	33.2 (0.0, 409.7)	14.0 (0.0, 291.0)	18.3 (0.0, 410.4)	368.0 (0.0, 1638.0)	519.0 (0.0, 2975.5)
4	8.0 (0.0, 32.0)	9.9 (0.0, 55.9)	38.0 (0.0, 256.0)	50.7 (0.0, 436.9)	22.0 (0.0, 273.0)	27.0 (0.0, 499.1)	707.5 (1.0, 1646.0)	900.9 (2.0, 3238.2)
5 (most deprived)	9.0 (0.0, 31.0)	10.8 (0.0, 42.9)	59.0 (1.0, 198.0)	77.4 (1.1, 367.2)	29.0 (0.0, 196.0)	36.8 (0.0, 333.3)	1060.0 (11.0, 1654.0)	1327.0 (12.1, 3040.4)

*Adjusted figures are per 1,000 population.

The median number of accessible facilities for vigorous intensity activities, both unadjusted and population adjusted, also increased with increasing deprivation from Q2 to Q5 for all modes of transport. Therefore, for both bicycle and car, it does not appear that there is a difference in the patterns observed for moderate and vigorous intensity activity facilities.

Similarly, for the 30 minute threshold (shown in Table 2) the median number of accessible facilities for vigorous intensity activity increased with increasing deprivation from Q2 to Q5 for all modes of transport. The median number of moderate intensity activity facilities accessible within 30 minutes followed a similar trend for all modes of transport.

**Table 2:** Median, minimum and maximum number of accessible facilities within 30 minutes by mode of transport and deprivation

**MODERATE INTENSITY FACILITIES**	**WALKING**		**CYCLING**		**BUS**		**CAR**	
**Income deprivation quintile**	Unadjusted	Adjusted	Unadjusted	Adjusted	Unadjusted	Adjusted	Unadjusted	Adjusted
1 (most affluent)	6.0 (0.0, 65.0)	7.9 (0.0, 116.7)	32.5 (0.0, 318.0)	43.3 (0.0, 595.3)	33.0 (0.0, 482.0)	42.5 (0.0, 892.4)	798.5 (3.0, 1341.0)	929.4 (4.3, 2326.6)
2	4.0 (0.0, 69.0)	5.9 (0.0, 119.3)	21.0 (0.0, 317.0)	28.4 (0.0, 586.5)	27.5 (0.0, 540.0)	35.5 (0.0, 809.5)	440.5 (0.0, 1459.0)	605.2 (0.0, 2648.9)
3 (middling)	6.0 (0.0, 67.0)	7.6 (0.0, 111.7)	25.0 (0.0, 318.0)	32.5 (0.0, 559.6)	31.0 (0.0, 511.0)	42.1 (0.0, 830.3)	542.0 (1.0, 1478.0)	710.3 (1.1, 2581.2)
4	8.0 (0.0, 63.0)	9.9 (0.0, 104.7)	34.0 (0.0, 325.0)	46.5 (0.0, 548.0)	45.0 (0.0, 475.0)	57.4 (0.0, 868.4)	852.0 (2.0, 1500.0)	1013.8 (2.7, 2795.3)
5 (most deprived)	8.0 (0.0, 56.0)	10.7 (0.0, 97.9)	55.0 (1.0, 289.0)	70.5 (1.3, 544.3)	63.0 (2.0, 387.0)	80.0 (2.3, 725.0)	923.0 (9.0, 1467.0)	1051.2 (9.2, 2436.9)
**VIGOROUS INTENSITY FACILITIES**								
**Income deprivation quintile**								
1 (most affluent)	11.0 (0.0, 82.0)	14.6 (0.0, 131.1)	65.5 (0.0, 415.0)	86.4 (0.0, 747.0)	57.0 (0.0, 626.0)	75.9 (0.0, 1147.4)	1332.5 (9.0, 2491.0)	1589.1 (12.8, 4468.2)
2	9.0 (0.0, 88.0)	11.2 (0.0, 143.9)	38.0 (0.0, 413.0)	48.7 (0.0, 761.3)	43.0 (0.0, 712.0)	58.4 (0.0, 1043.7)	790.5 (0.0, 2642.0)	1048.1 (0.0, 4780.5)
3 (middling)	12.0 (0.0, 81.0)	15.3 (0.0, 111.9)	44.0 (0.0, 417.0)	56.8 (0.0, 734.7)	53.0 (0.0, 669.0)	70.8 (0.0, 1132.1)	965.0 (0.0, 2686.0)	1282.2 (0.0, 4690.8)
4	15.0 (0.0, 68.0)	19.2 (0.0, 118.9)	67.5 (0.0, 408.0)	89.2 (0.0, 712.0)	80.0 (0.0, 676.0)	105.6 (0.0, 1129.8)	1441.5 (2.0, 2719.0)	1895.6 (4.0, 5007.8)
5 (most deprived)	19.0 (0.0, 69.0)	24.2 (0.0, 101.7)	125.0 (1.0, 380.0)	153.3 (1.4, 715.6)	135.0 (3.0, 584.0)	170.5 (4.4, 952.9)	1909.0 (11.0, 2669.0)	2156.4 (13.1, 4593.2)

*Adjusted figures are per 1,000 population.

### Distribution of facilities by area-level deprivation and urbanicity

**Figure 1:**
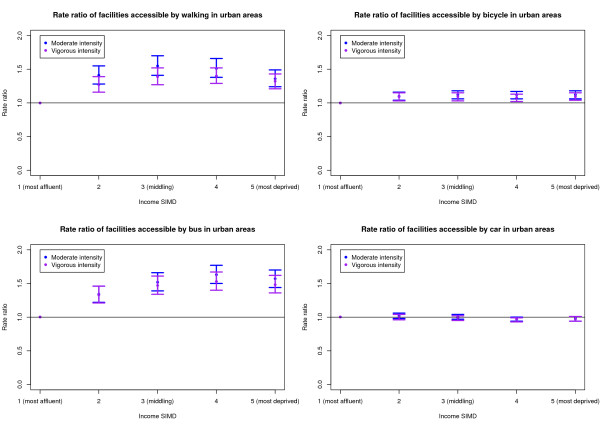
Rate ratio of facilities accessible within 20 minutes of urban areas by deprivation and mode of transport.

**Figure 2:**
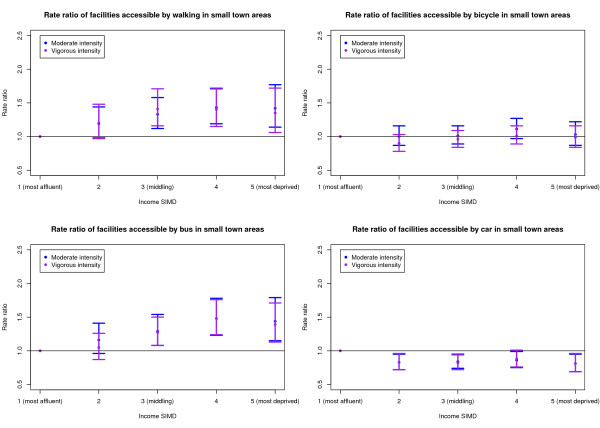
Rate ratio of facilities accessible within 20 minutes of small town areas by deprivation and mode of transport.

**Figure 3:**
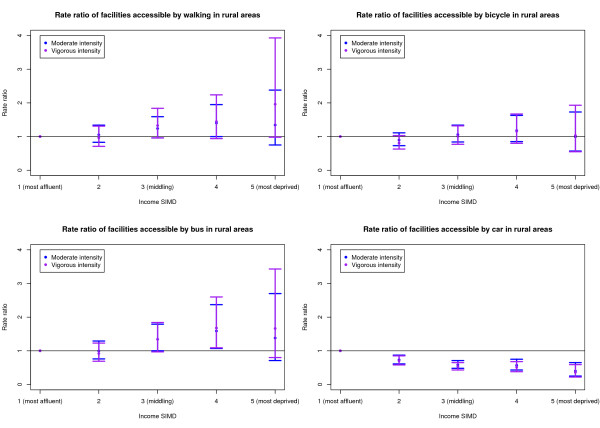
Rate ratio of facilities accessible within 20 minutes of rural areas by deprivation and mode of transport.

Of the 6,412 DZs considered, 4,473 (69.8%) were classified as urban areas, 826 (12.9%) as small town and 1,113 (17.4%) as rural areas. The multilevel modelling was carried out for urban, small town and rural areas separately for both the 20 and 30 minute journey time thresholds. Since the patterns in the results were similar for both the 20 and 30 minute thresholds, only the 20 minute threshold models are presented in this paper. Figures 1 to 3 show the RRs and 99% confidence intervals for the moderate and vigorous intensity activity facilities by Income SIMD for urban, small town and rural areas respectively.

### Urban areas

Considering the walking, cycling and bus accessibility models, Q2 (the second most affluent quintile) to Q5 (most deprived) had significantly higher RRs of both accessible moderate intensity activity facilities and accessible vigorous intensity activity facilities than Q1. No significant differences were identified in the number of accessible moderate intensity activity facilities by car between any of the Income SIMD quintiles. However, Q4 was shown to have a significantly lower RR of vigorous intensity activity facilities than Q1.

### Small towns

Income SIMD quintiles 3 to 5 had significantly higher RRs of both accessible moderate and accessible vigorous intensity activity facilities than Q1 for the walking and bus models. No significant differences were identified between quintiles for the cycling accessibility model for either the moderate or vigorous intensity activity facilities. By car, Q1 had a significantly higher RR than Q2 to Q5 for the moderate intensity activity facilities, whilst Q1 had a significantly higher RR of vigorous intensity activity facilities than Q2, Q3 and Q5.

### Rural areas

In rural areas, Q4 had a significantly higher RR of accessible moderate intensity activity facilities than Q1 for both the walking and the bus model and a significantly higher RR of accessible vigorous intensity activity facilities than Q1 by bus. There was no evidence of a difference in RR of accessible vigorous intensity activity facilities by walking and no significant differences were identified between quintiles for the cycling model for either moderate or vigorous intensity activity facilities. Q1 had a significantly higher RR than all other quintiles when travelling by car for both the moderate and vigorous intensity activity facilities.

## Discussion

In general, the modelling results suggest that those living in the most affluent neighbourhoods have poorer access to facilities for moderate and vigorous intensity physical activity on foot, by bicycle and by bus than those in less affluent areas. This is in agreement with our previous analyses of the accessibility of all facilities by these modes of transport, in which more affluent areas were found to have poorer accessibility [16,17], and concurs with previous national studies identifying reduced access in more affluent areas [9]. On the other hand, the results constrast national studies which identified greater access in the more affluent areas [8,10] or found no significant differences in access by area [12] but it is difficult to directly compare studies due to differences in the definition of access, the spatial scales and the measures of area level deprivation adopted. However, the pattern in our modelling results appears to differ for those with access to a car, as the most affluent areas appear to have a higher number of facilities for both moderate and vigorous physical activity accessible by car than the more deprived. This pattern was also observed, particularly in rural areas, in our previous analysis examining all, public and private facilities accessible by car [17]. It therefore appears that having access to a car in more affluent areas may help to counteract the generally disadvantageous access experienced in those areas. While this may be of benefit to individuals and households with access to a car, it highlights the possibility that those without cars living in more affluent areas may be more disadvantaged in terms of access to facilities than if they were living in areas of greater aggregate deprivation.

### Strengths and limitations

This analysis adds to current literature on the accessibility of PA facilities by categorising the facilities according to the likely physical intensity of the activities typically undertaken in such facilities. However, it is important to note that although each facility was assigned only one MET value, it is possible for a wide range of activities to be undertaken at some facilities or for different individuals to participate in the same activity at different levels of intensity. For example, while it is possible to exercise at vigorous intensity in a swimming pool, it is also possible to use a swimming pool for only light or moderate intensity activity. As this was an ecological analysis and it was not possible to identify exactly how each facility was used in practice, we considered it reasonable to assign each facility to a single intensity category and, in the case of facilities such as church halls, to make a conservative assumption that those would be used for moderate intensity activities.

One of the key strengths of this analysis is the use of transport networks which allow the opportunity to compare the accessibility of facilities across various modes of transport. However, it is important to note that, for simplicity, the transport network did not take into account the topography or route quality and that average walking and cycling speeds and estimated free flow speeds for cars were used to estimate the number of accessible facilities. A further limitation is that the number of accessible facilities was assessed from the population weighted centroid, and whilst DZs are the smallest available measure of area used in Scotland they do vary in size. Therefore, it may not be realistic to assume that the travel time from the population weighted centroid is truly representative for residential locations within the DZ.

A further strength of this analysis was that we examined more than one time threshold. Although only the modelling for the 20 minute threshold was reported in this paper, the trends and modelling results showed similar patterns for the 30 minute time threshold. A point to note, however, is that in some cases statistically significant results for the 20 minute threshold were no longer significant for the 30 minute threshold. Therefore, future work should focus on examining the amount of time people are willing to spend travelling to fixed physical activity facilities in order to determine the most appropriate threshold to apply for each mode of transport. Work already undertaken on this topic has shown that distances travelled for recreational physical activity purposes varies by demographic characteristics, destination type and the activity undertaken at the destination, with greater travel distances reported for those participating in moderate intensity rather than vigorous intensity activities [36].

Having said that, the number of facilities that can be reached within a certain time threshold by different modes of transport may not reflect true accessibility. Facilities may not be truly accessible for a number of reasons, for example because they are located in an undesirable neighbourhood or, as suggested by a qualitative study of the use of urban green space in Scotland, because they are perceived to be ‘not for me’ [37]. Giles-Corti et al. report that although residents living in areas of low socioeconomic status in Perth, Australia had equal or better access to recreational physical activity facilities, they were less likely to use facilities which charged an entrance fee even after taking household income into account, suggesting that access alone does not determine use [5]. It may not be access to fixed PA facilities which is the key factor in aiding individuals to meet PA recommendations. Our study does not include information about green space or other spaces where PA can be undertaken free of charge. In Australia, Bauman et al. found proximity to the coast was associated with a higher likelihood of participating in adequate activity [38] whilst other studies have shown that the most popular forms of physical activity include walking and gardening [39], which are not typically undertaken in facilities of the type examined in our analysis. This emphasises the need to include other activity spaces, not just simply fixed recreational PA facilities, in a comprehensive analysis of the accessibility of opportunities for physical activity.

Our next step will be to explore the extent to which the area level accessibility of fixed physical activity facilities by different modes of transport is linked to individual physical activity levels and obesity.

## Abbreviations

DZ: data zone; GIS: Geographic Information System; MET: Metabolic equivalent of task; PA: Physical activity; RR: Rate ratio; SIMD: Scottish Index of Multiple Deprivation.

## Competing interests

The authors declare that they have no competing interests.

## Authors’ contributions

AE, DO and NSF devised the study, JM and KEL performed the statistical analysis, NSF mapped the facilities and NSF and YW constructed the transport networks. All authors contributed to the drafting of the manuscript and approved the final version.
